# Antibiotic sensitivity profile of *Staphylococcus aureus* isolated from HIV/AIDS patients presenting with pyoderma, at the Yaounde Central Hospital, Cameroon

**DOI:** 10.11604/pamj.2020.37.185.14595

**Published:** 2020-10-27

**Authors:** Michel Kengne, Hermann Brice Nkuinzeu Fotie, Julius Mbekem Nwobegahay, Patrick Njukeng Achiangia, Ublad Tamoufe, Daniel Ter Goon, Junior Bitoungui Mboua, Gladys Tchanana, Akemfua Fualefac, Hilda Echelibe, Rodine Kouonang Djonkam, Awung Nkeza

**Affiliations:** 1 Department of Microbiology/Immunology, School of Health Sciences, Catholic University of Central Africa, Yaounde, Cameroon,; 2 Military Health Research Center (CRESAR), Yaounde, Cameroon,; 3 Faculty of Science, University of Dschang, Dschang, Cameroon,; 4 METABIOTA, Yaounde, Cameroon,; 5 Department of Nursing Sciences, University of Fort Hare, Alice, South Africa,; 6 Yaounde Central Hospital, Yaounde, Cameroon

**Keywords:** Human immunodeficiency virus, *Staphylococcus aureus*, antibiotics, sensitivity, pyoderma

## Abstract

**Introduction:**

the purpose of this study was to evaluate the in vitro activity of several antibiotics against strains of Staphylococcus aureus isolated from pyoderma in people living with Human Immunodeficiency Virus (HIV), consulting at the day clinic of the Yaoundé Central Hospital.

**Methods:**

this was a prospective, cross-sectional study which was carried out in five months (November 2013-March 2014). Fifty-three (53) pus specimens were collected; from which the isolation of Staphylococcus aureus was made using Chapman agar. Mannitol fermentation, catalase, coagulase and DNase tests were used for species identification. Antibiotic sensitivity of each strain was determined by the agar diffusion method.

**Results:**

forty-eight (48) strains of Staphylococcus aureus were isolated (90.56%). A high rate of sensitivity to antibiotics was observed in many strains: vancomycin (100.0%), pristinamycin (100.0%), chloramphenicol (100.0%), oxacillin (97.9%), cefoxitin (97.9%), gentamicin (87.5%), tobramycin (83.3%). However, some strains had strong resistance to penicillin G (89.6%) and cotrimoxazole (64.6%). The proportion of Methicilin Resistant strains of Staphylococcus aureus (MRSA) was low (2.0%). The kanamycin-tobramycin-gentamycin phenotype (KTG) was most common in the aminoglycosides resistant strains; the same as the induced phenotype E stains (iMLSB) in macrolides resistant strains. **Conclusion:** these results indicate that many of these antibiotics tested are still effective against strains of Staphylococcus aureus.

## Introduction

Infection with the Human Immunodeficiency Virus (HIV) has a great impact on skin diseases [1] and several HIV patients have dermatitis at some point during their illness [2]. The outer part of the skin can be the source of opportunistic infections (viral, bacterial, fungal and parasitic) in patients whose immune defense has been impaired. Bacterial infections are a common cause of morbidity in HIV-infected patients with a much higher incidence than in the general population. Whatever the stage of HIV infection, the skin is frequently affected by often minor and easily treated lesions, but they are sometimes indicative of more severe complications [3]. Pyodermas are skin infections caused by pyogenic bacteria and they can progress to suppuration. *Staphylococcus aureus* and *Streptococcus pyogenes* are the most implicated in these purulent infections [4]. *Staphylococcus aureus*, usually found in infections progressing to suppuration, is a ubiquitous bacterium endowed with a remarkable ability to survive in an adverse environment. These staphylococcal infections weaken the immune system of people living with HIV/AIDS (PLWHA). It is therefore a significant nuisance for many patients.

Faced with these suppurative infections, β-lactams, aminoglycosides and macrolides are generally considered major anti staphylococcal, alongside other antibiotics which are also used in the treatment of these infections (fusidic acid, cotrimoxazole and fluoroquinolones) [5]. But the unpredictable evolution of resistance, particularly in our region where the pressure of drug selection is very high, should lead to regular monitoring of the sensitivity of the bacterial species to various antibiotics. In Cameroon, data on antibiotic susceptibility to *Staphylococcus aureus* responsible for pyoderma are rare. Immunodepression being one of the factors that may contribute to bacterial resistance, it is important to assess the susceptibility profile of pathogen isolated from suppurative infections in PLWHA and determine some resistance phenotypes.

## Methods

This was a prospective cross-sectional study over a period of five months (November 2013-March 2014). Ethical clearance was obtained from the National Ethics Committee for Human Health Research (N^0^2014/11/515/CE/CNERSH/SP) in Yaounde, Cameroon. This study focused on pyoderma seen in patients with HIV/AIDS in the day care clinic at the Yaoundé Central Hospital (HCY). The different forms of pyoderma were photographed and subsequently identified by a dermatologist.

Pus specimens were collected from patients of both sexes who were not exposed to antibiotics within the previous fifteen days. All strains were identified by conventional bacteriological methods: isolation on agar, Chapman, direct examination by gram stain, mannitol fermentation, production of catalase, coagulase (agglutination test with SlidexstaphBioMerieux and rabbit-plasma coagulation test) and thermonuclease testing. The sensitivity of the various strains was determined using penicillin G (10IU), cefoxitin (30μg), oxacillin (1μg), gentamicin (10IU), tobramycin (10IU), kanamycin (30μg), pefloxacin (5μg), rifampin (5μg), fosfomycin (50μg), cotrimoxazole (25μg), vancomycin (30μg), tetracycline (30IU), lincomycin (15μg), pristinamycin (15μg), erythromycin (15μg), fusidic acid (30μg) and chloramphenicol (30μg).

The study of the sensitivity of the strains was performed by the method of agar diffusion using Mueller-Hinton, as recommended in 2013 by the Committee on Antibiotic susceptibility of the French Society for Microbiology (CA-SFM) [6]. Methicillin resistance was investigated using a cefoxitin disk (30mcg); in addition to oxacillin disk, added onto hyper saline media previously inoculated with a dense inoculum of approximately 107 UFC/ml and incubated for 24 hours at 37°C [7]. Any strains with a diameter of inhibition less than 27mm around cefoxitin on hyper saline Mueller-Hinton plates were declared resistant to methicillin (MRSA). Quality control was done using strain ATCC25923. Data were entered and analyzed using SPSS version 12.0 for windows (SPSS, Inc., Chicago, IL). Discrete variables were expressed as percentages and frequencies.

## Results

Fifty-three (53) samples were collected from patients presenting with pyoderma. The mean age of patients was 40.34 years (range 26-60 years). There were 16 (30.2%) men and 37 (69.8%) women. Forty-eight strains of *Staphylococcus aureus* were isolated (90.57%). Abscesses 19 (36.0%) and boils 17 (32.0%) were the two types of pyoderma most encountered. The type of pyoderma least encountered was paronychia (whitlow) (9.0%) as shown in Table 1. A high rate of sensitivity to antibiotics was observed in many strains (Table 2): vancomycin (100.0%), pristinamycin (100.0%), chloramphenicol (100.0%), oxacillin (97.9%), cefoxitin (97.9%), gentamicin (87.5%), tobramycin (83.3%). However, some strains displayed a high rate of resistance to penicillin G (89.6%) and cotrimoxazole (64.6%). The proportion of Methicilin Resistant strains of *Staphylococcus aureus* (MRSA) was low (2.0%). Three resistance phenotypes were observed among the aminoglycosides resistant isolates and three within the macrolides resistant isolates (Table 3, Table 4). Among the 13 strains isolates resistant to aminoglycosides, 6 were KTG phenotype and 2 strains were KT phenotype. Among macrolides resistant isolates, 8 strains were phenotype E and 1 was phenotype L.

**Table 1:** types of pyoderma in the study population

Pyoderma	Frequency (%)
Abscess	19 (36.0%)
Boils	17 (32.0%)
Folliculitis	12 (23.0%)
Whitlow	5 (9.0%)
Total	53 (100%)

**Table 2:** percentage of sensitivity of *Staphylococcus aureus* isolates to antibiotics

Antibiotics		Strains (n=48)	
		**Sensitive (S)**	
		**Frequency**	**%**
β-lactamines	Penicillin	5	10.4
	Oxacillin	47	97.9
	Cefoxitin	47	97.9
Aminosides	Gentamycin	42	87.5
	Kanamycin	34	72.9
	Tobramycin	39	83.3
Macrolides	Erythromycin	36	75.0
	Lincomycin	43	89.6
	Pristinamycin	48	100.0
Phenicols	Chloramphenicol	48	100.0
Cyclines	Tetracyclines	27	56.3
Fluoroquinolones	Pefloxacin	34	72.9
Glycopeptides	Vancomycin	48	100.0
Sulfamides	Cotrimoxazole	16	35.4
Others	Fusidic acid	42	87.5
	Rifampicyn	44	91.7
	Fosfomycin	39	81.3

**Table 3:** resistance mechanism/phenotype of the strains to aminoglycosides

Kanamycin	Tobramycin	Gentamycin	Phenotypes	Mechanism	Strains (n=48)
S	S	S	Wild type	Sensitive	35 (72.9%)
R	S	S	K	APH(3')-III	5 (10.4%)
R	R	S	KT	ANT(4')	2 (4.1%)
R	R	R	KTG	APH(2'')-AAC(6')	6 (12.5%)

R: resistant; S: sensitive; K: kanamycin; KT; kanamycin-tobramycin; KTG: kanamycin-tobramycin-gentamycin; APH: O-Phosphotransferases; ANT: O-Adenyltransferases; AAC: N-Acetyltransferases

**Table 4:** resistance mechanism/phenotype of the strains to macrolides

Eryhromycin	Lincomycin	Pristimycin	Phenotypes	Mechanism	Strains (n=48)
S	S	S	Wild type	Sensitive	35 (72.9%)
R	S	S	E	Inducible MLSB	8 (16.6%)
R	R	S	EL	Constitutive MLSB	4 (8.3%)
S	R	S	L	Efflux	1 (2.0%)

E: erythromycin; EL: erythromycin-Lincomycin; MLSB: macrolide-lincosamide-streptogramin B

## Discussion

The knowledge and surveillance of the sensitivity profile of *Staphylococcus aureus* strains to commonly used antibiotics are important for the treatment of infections caused by this bacterial species. It is important for institutions to conduct periodic assessments of the susceptibility of isolates of *Staphylococcus aureus* to antibiotics currently in use. Our study addresses this goal by analyzing the in vitro activity of seventeen antibiotics against 48 strains of *Staphylococcus aureus*. Of the 53 samples collected, 48 (90.56%) strains of *Staphylococcus aureus* were isolated. This result is higher than that (78.5%) reported in 2003 by Lorette *et al*. [8]. Of the 48 strains, obtained, 97.9% were sensitive to oxacillin and cefoxitin, with a resistance rate of 2.1%. This low rate is similar to that (1.9%) reported in Morocco in 2001 by Belabbès *et al*. [9] and in 2015 in Spain by Imaz *et al*. [10] who found a rate of 1% and 2% respectively in nasal and pharyngeal samples respectively. On the other hand, a strain resistant to oxacillin, expressed inhibition of less than 27mm around the 30μg cefoxitin disk and it was declared as Methicilin Resistant *Staphylococcus aureus* (MRSA). The multidrug-resistant nature of this MRSA was confirmed by resistance to β-lactams (cefoxitin, oxacillin and penicillin) and several other antibiotics (gentamicin, gerythromycin, pefloxacin and cotrimoxazole).

Clinically, these antibiotics are commonly used by patients; sometimes without any prescription. This habit of auto medication could partly explain the multi-resistant nature of this bacterial species as self-medication is the most common reason for the development of human pathogen resistance to antibiotic drugs due to selective pressure [11]. It is also well established that MRSA carries a staphylococcal cassette chromosome mec (SCCmec) that is composed of the mec gene complex encoding methicillin resistance (expression of penicillin binding protein PBP2a that has a low affinity for β-lactam antibiotics) and the ccr gene complex that encodes recombinases responsible for its mobility. These elements also carry various resistance genes for non-beta-lactam antibiotics. Consequently, after acquiring an SCCmec element, MRSA undergoes several mutational events and evolves into the most difficult-to-treat pathogen in hospitals, against which all extant antibiotics including vancomycin are ineffective [12]. In this study, 43 (89.6%) strains of *Staphylococcus aureus* were resistant to penicillin G. This is most likely as a result of wide use of this antibiotic in Cameroon. Penicillinase production is also highly responsible for resistance to Penicillin G [13]. This finding is similar to that previously reported in 2009 by Elhamzaoui *et al*. [14] with a resistance rate of 90% and to that of Sampane-Donkor *et al*. [15] with a resistance rate of 100%. However, this result is higher than that (62.96%) reported in 2013 in Algeria by Chaalal [16].

Our results further showed that there was no vancomycin-resistant strain. This result is consistent with that reported in 2010 by Elazhari *et al*. [17] as well as that reported in 2013 by Chaalal [16]. A high resistance rate of the strains to fusidic acid was noted (87.5%). This finding contrasts with that reported in Casablanca by Belabbès *et al*. (2001), where a result of 4.5% was recorded. The sensitivity rate of 72.9% and 56.3% obtained for pefloxacin and tetracycline respectively were different from those reported in 2010 by Elazhari *et al*. [17]. The decrease in sensitivity of the isolates to these agents could be due to the fact that fluoroquinolones in general and cyclins are available in our country and are most often used inappropriately without prescription. Among the 48 strains isolated, 44 (91.7%) were sensitive to rifampicin, with a resistance rate of 8.3%. Fosfomycin was active for nearly 81.3% of the total strains, although this rate is lower than that reported in 2010 by Elazhari *et al*. [17], where all the isolated strains were sensitive to this antibiotic. Macrolides, lincomycin and streptogramin (MLS) have always seemed to be good antistaphylococcal agents. This study revealed the sensitivity rates of 75.0%, 89.6% and 100.0% for erythromycin, lincomycin and pristinamycin respectively. The rates of sensitivity to lincomycin and erythromycin were lower than those found in 2010 (98.7% and 93.4%) respectively by Elazhari *et al*. [13]. All the isolates were susceptible to Pristinamycin. A similar result was obtained in 2003 by Lorette *et al*. [8].

*Staphylococcus aureus* strains displayed a good sensitivity rate to aminoglycosides namely gentamicin 87.5%, tobramycin 83.3% and kanamycin 72.9%. A similar result was also found in Morocco in 2010 by Elazhari *et al*. [17]. A low sensitivity (35.4%) was observed with cotrimoxazole which is much higher than that reported in 2010 by Elazhari *et al*. [17]. The increased resistance of the strains to cotrimoxazole in our study could be explained by the fact that the study participants consisted of people living with HIV, with most of them on antiretroviral treatment. Given that cotrimoxazole is often associated with greater access to antiretroviral treatment to combat opportunistic bacterial infections such as pneumococcal disease, its wide use might have resulted to the high resistance rate obtained (64.6%).

In our study, the K phenotype was identified in 5 strains (10.4%). Phenotype KT was identified in 2 strains (4.2%) and KTG phenotype was identified in six strains (12.5%). These three phenotypes were reported in 2010 by Elazhari *et al*. [17] but at different rates of resistance (1.3%, 1.3% and 8.2% respectively for the KTG, KT and K phenotypes).

## Conclusion

These results indicate that many of these antibiotics tested are still effective against strains of *Staphylococcus aureus*, although some strains have developed strong resistance to penicillin G.

### What is known about this topic

- Many HIV-positive patients are given primary (thrimethoprim/sulphamethoxa zole) prophylaxis and exposure to this antibiotic might increase the risk of antibiotic resistance in a variety of bacterial pathogens that may infect this high risk population.

### What this study adds

- Vancomycin, pristinamycin, chloramphe nicol, oxacillin, cefoxitin, gentamicin and tobramycin are the appropriate antibiotics for treating pyoderma found in HIV/AIDS patients in the study set up.
